# The Indian Medical Congress at Calcutta—The Fevers of India—The National Health Society

**Published:** 1895-02-02

**Authors:** Ernest Hart


### LETTERS FROM INDIA.

### THE INDIAN MEDICAL CONGRESS AT CALCUTTA —THE FEVERS OF INDIA — THE NATIONAL HEALTH SOCIETY.

Communicated by Mrs. Ernest Hart.

India is associated in the mind, both of the medical and the lay public, with two great diseases—fever and cholera. Among so many admirable papers read at this congress, it will not be, I trust, thought invidious to select for special mention those of Dr. Alexander Crombie, on the "Fevers of India," and of Dr. Haffkine on "Inoculation Against Cholera," as being of the deepest interest at the present time.

Dr. Alexander Crombie, Surgeon-Lieut.-Colonel, Indian Medical Service, and President of the Medical Section, drew attention to the significant fact that while in 1892 deaths from cholera in India were estimated to amount to 750,000, those from fevers reached the high figure of 4,500,000.

Indian fevers Dr. Crombie divides into two great groups—malarial and non-malarial. In the first, quinine is the prophylactic, in the second it is useless. Speaking of remittent fevers, which are far the most fatal in India, Dr. Crombie distinguished between the malarial and the non-malarial forms. There is little room to doubt that the former are produced by similar amœbic forms to those which produce the intermittent fevers, and that malarial remittent fevers are in fact intermittent fevers, in which either the pyrexial stage is unduly prolonged, owing to the excessive toxic qualities of the organism, so that a new accession of fever comes on before the previous attack has entirely subsided, or they are cases in which there are at one and the same time two sets of organisms in the blood whose cycles of life are parallel, but not synchronous, thus keeping up a continous condition of pyrexia. Dr. Crombie urged that while the demonstration of the malarial parasite in the blood of the finger may be regarded as conclusive of the malarial nature of the case, failure to find it could in no way justify the opposite conclusion in the face of distinct clinical symptoms of a malarial type. Diagnosis is important, because treatment hinges upon it. In malarial remittent fever quinine is the antidote, and should be administered in large doses, and in the early stages of the disease physicians should not shrink from giving from 60 to 100 grains in the twenty-four hours. It is in the early period of the growth of the amœba, while still unpigmented, and before segmentation, that quinine has such a deterrent effect. When it ceases to grow and prepares for segmentation and sporulation, that is just before the rigor and new accession of fever, quinine has little or no effect upon it, and Dr. Crombie, therefore, warmly deprecated the view that quinine can only be safely given during the period of a pyrexia.

There has arisen in India, among natives, a disbelief in the prophylactic power of quinine, but Dr. Crombie emphatically declares that once assured the fever is malarial, quinine cannot be given too freely.

On the other hand, quinine in non-malarial continued fevers is not only of no avail, but is sometimes distinctly harmful. Dr. Crombie is of the opinion that the non-malarial fevers, many of which are typhoid, and many more resemble that disease, although of quite a different nature, are more common than the malarial. Simple continued fever ushered in with rigor and sudden rise of temperature, with defervescence on the 14th, 21st, or 28th day, is easily mistaken for typhoid; but Dr. Crombie contends that simple continued fever is an entity distinct from typhoid, and is due to some other organism than the bacillus of Eberth. Non malarial remittent fever attacks Europeans occasionally, but is essentially a disease of the native. Its pathogenic organism is yet to be discovered, and here there is a fruitful field for inquiry.

[Dr. Haffkine's address gave a full account of the work he has done up to the present time; but, as we have already published some of these results as they have been announced, it does not appear necessary just now to repeat them. Certainly the inoculations seem not to have been without influence; but whether the effect has been more than a very transient antagonism is a question for the decision of which the data do not at present appear sufficient.]

While Dr. Haffkine is advocating one method of protection against cholera, Mr. Hart is advocating another. Mr. Hart's has certainly the advantage of simplicity. It is contained in four words—"Boil your drinking water." Water undoubtedly conveys the contagium of cholera and malaria; therefore boil it before drinking, and kill the microbes. At a very representative and influential meeting convened by Lady Elliott at Belvedere to found a branch of the National Health Society in Calcutta, Mr. Hart explained that if by means of fly-sheets and pictures the people could be taught "the policy of the tea-kettle," and to boil all their drinking water, the ravages of cholera and malaria would be greatly diminished. This meeting, which was called at Mr. Hart's suggestion, was one of the most successful ever held for such a purpose in Calcutta. Sir Charles Elliott, the Lieutenant-Governor, was in the chair, and there were present Dr. Harvey, Dr. Simpson, Dr. Joubert, Miss Staley, M.B.Lond. (of Delhi), a large number of Hindu maharajahs and rajahs and Mohammedan nawabs and princes in splendid costumes, English ladies well known in society, native judges, barristers, and Brahmo-Somag ladies in their graceful draperies. About 4,000 rupees were subscribed on the spot. An influential working committee was appointed to carry on the health propaganda.

Calcutta, January 4th.

